# Main Diagnostic Criteria Usually Does Not Work for Autoimmune Pancreatitis Wrongly Presuming Malignancy

**DOI:** 10.1155/2023/6652881

**Published:** 2023-09-29

**Authors:** Lei Gong, Bin Shu, Fei Yu, Xinjing Zhang, Jianfei Chen, Jirun Peng

**Affiliations:** ^1^Department of Surgery, Beijing Shijitan Hospital, Capital Medical University, Beijing 100038, China; ^2^Center of Hepatopancreatobiliary Diseases, Beijing Tsinghua Changgung Hospital, School of Clinical Medicine, Tsinghua University, Beijing 102218, China

## Abstract

**Background:**

Autoimmune pancreatitis (AIP) usually responds dramatically to steroid therapy. Occasionally, however, misdiagnosed patients have undergone pancreaticoduodenectomy. This study is aimed at providing useful information to improve the accuracy of diagnosis before surgery and thus avoid unnecessary resections in patients with AIP.

**Methods:**

From January 2015 to February 2020, a series of patients were enrolled, having undergone pancreaticoduodenectomy for presumed malignancy. AIP diagnoses were confirmed by postoperative pathology. The demographic and clinical data of the AIP patients were evaluated. The main diagnostic criteria (HISORt, Asian, and ICDC) for AIP were applied to assess whether and how unnecessary surgery could have been avoided.

**Results:**

A total of 124 cases of pancreaticoduodenectomy were performed for presumed malignancy. Six patients were diagnosed with benign disease and five with AIP. The prevalences of benign disease and AIP were 4.8% and 4%, respectively. Four patients were female and 1 male, with a mean age of 60.0 years old. Jaundice, pain, and weight loss were observed in 100%, 20%, and 40% of AIP patients, respectively. The radiologic features of the AIP patients were a diffusely enlarged gland (40.0%), a focally enlarged gland (40.0%), pancreatic ductal dilatation (60.0%), upstream parenchymal atrophy (20.0%), bile duct thickening (66.0%), and bile duct stricture (40.0%). Based on the diagnostic criteria for AIP, surgery could have been avoided in two cases.

**Conclusions:**

IgG4 measurement and integrated use of major diagnostic criteria should be emphasized in every patient eligible for pancreaticoduodenectomies.

## 1. Introduction

Pancreaticoduodenectomy (PD) is one of the most complex procedures in general surgery. Indications for PD include pancreatic head cancer, periampullary carcinoma, and distal bile duct carcinoma. As surgical techniques and perioperative management have continuously improved, mortality from PD has declined over time. However, reported mortality is still 4% to 8% in population-based studies [1, 2], and as low as 1% only in high-volume centers [3, 4]. The complication rate of PD is as high as 22% to 41% and has not declined significantly over time [5]. Moreover, occasionally, the postoperative pathology workup reveals benign disease in patients who underwent PD for presumed malignant disease. The incidence of benign disease after PD is reportedly 6% to 9.9% [6–8]. Among these patients, autoimmune pancreatitis (AIP) is one of the most frequent benign diseases diagnosed after PD [9].

AIP is an autoimmune disease clinically characterized by obstructive jaundice with or without a pancreatic mass and therapeutically characterized by a dramatic response to steroids [10]. Two types have been identified: type 1 and type 2. Various diagnostic criteria for AIP have been proposed by researchers from Asia, Europe, and North America [11–13]. Nevertheless, the correct diagnosis of AIP before surgery remains a challenge for surgeons. Preoperative misdiagnosis may lead to unnecessary resections.

This study is aimed at evaluating the prevalence of benign diseases, especially AIP, in patients who underwent PD for presumed malignancy in a tertiary hospital in mainland China. The clinical characteristics and treatment courses of these patients were carefully reviewed, based on the main diagnostic criteria for AIP, to assess whether and how unnecessary surgery could have been avoided in these patients.

## 2. Methods

### 2.1. Patients and Data Collection

From January 2015 to February 2020, consecutive patients who underwent PD for presumed malignancy at the Department of Hepatopancreatobiliary Surgery of our hospital were included in this observational study.

Demographic characteristics (age and gender) and mortality were recorded for all patients. Clinical characteristics (jaundice, pain, weight loss, diabetes, chronic pancreatitis, autoimmune disease, smoking, and alcohol consumption) and imaging characteristics (an expert radiologist blinded to the study reevaluated the imaging) were evaluated for AIP patients. The HISORt [14] and Asian [15] diagnostic criteria and the international consensus diagnostic criteria (ICDC) [16] were applied to assess whether and how unnecessary surgery could have been avoided in these patients.

## 3. Results

From January 2015 to February 2020, 124 patients received PD for presumed malignancy (pancreatic head cancer, periampullary carcinoma, distal bile duct carcinoma, and others) in our department, of which 6 patients eventually received a postoperative diagnosis of benign disease. The ultimate pathologies of the six benign PDs were chronic pancreatitis in one case and AIP (type 1) in five cases. All five AIP patients were pathologically diagnosed as type 1 AIP with at least 3 of the following features: periductal lymphoplasmacytic infiltrate without granulocytic infiltration, obliterative phlebitis, storiform fibrosis, and more than 10 cells/HPF (high power field) IgG_4_ (immunoglobulin) positive cells. There was no significant difference in age or gender between patients with malignant compared with benign disease. The mortality was 4.8% (6/124) in all patients, 5.0% (6/118) in those with malignant disease, and 0% (0/6) in those with benign disease. Overall, the prevalence of benign disease and AIP was 4.8% and 4.0%, respectively, in all PD patients. AIP accounted for most (83.3%) of the benign diseases treated with PD.

The demographic and clinical characteristics of patients with AIP are shown in Table 1. Four patients were male and one female. The mean age was 60.0 years. Jaundice, pain, and weight loss were observed in 100%, 20%, and 40% of AIP patients, respectively. One patient had an underlying autoimmune disease, and another patient had a history of smoking. All 124 patients underwent CT (computed tomography) scans, with MRI (magnetic resonance imaging) in 101 cases, EUS (endoscopic ultrasound) in 12 cases, and ampullary biopsy in 5 cases. The imaging characteristics and preoperative radiologic diagnoses of AIP patients are listed in Table 2. The radiologic features of the AIP patients were a diffusely enlarged gland (40.0%), a focally enlarged gland (40.0%), pancreatic ductal dilatation (60%), upstream parenchymal atrophy (20%), bile duct wall thickening (60%), and bile duct stricture (40%). The preoperative radiologic diagnoses were distal cholangiocarcinoma in two patients, ampullary carcinoma in one patient, pancreatic cancer (PaC) in one patient, and neuroendocrine tumor in one patient.

All five patients recovered uneventfully postoperatively and were discharged successfully. Jaundice began to gradually disappear right after the surgery in all patients except one. In this patient, serum bilirubin continued to rise in the first 3 days postoperatively and gradually decreased after steroid therapy was given on the fourth day after surgery. This patient was discharged 33 days after surgery with a serum bilirubin of 60 *μ*mol/L and received steroid therapy for another 2 weeks. All the patients were followed up without recurrence of jaundice as of this writing.

**Figure 1:**
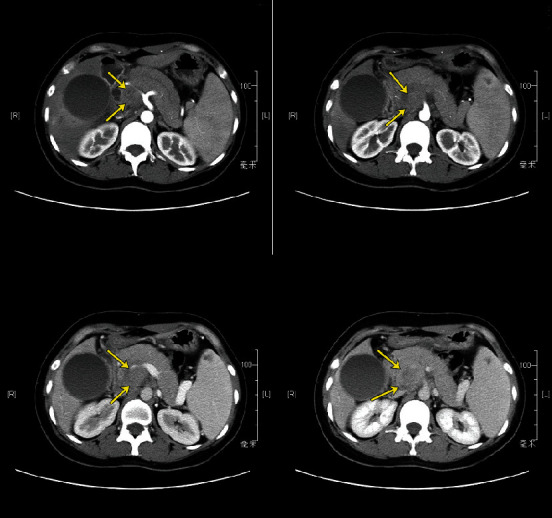
Indeterminate imaging. CT shows a diffusely enlarged gland with a low-density mass (arrows marked) adhering tightly to the superior mesenteric artery (SMA) and invading the superior mesenteric vein/portal vein (SMV/PV).

As shown in Table 3, one patient had elevated IgG_4_; one patient had a typical (seemingly diffusely enlarged gland) and one indeterminate (diffusely enlarged gland with low-density mass, Figure 1); only this patient underwent biopsy, EUS-FNA (endoscopic ultrasonography-guided fine needle aspiration), which was negative for AIP) imaging for AIP. Based on the HISORt, Asian, and ICDC diagnostic criteria for AIP, none of the five patients could have been definitely diagnosed with AIP even retrospectively, but surgeries could possibly have been avoided in two cases.

## 4. Discussion

In the current study, Asian, HISORt, and ICDC criteria were applied to assess whether and how unnecessary surgery could have been avoided in AIP patients. A reassessment of the clinical records of the five patients revealed that none of them could have been diagnosed definitively and correctly before surgery. However, had the preoperative workup been more comprehensive, unnecessary surgery could have been avoided in 40% (2/5) of the patients with AIP.

In patient no. 1, according to the HISORt criteria and ICDC, the elevation of serum IgG_4_ (<2-fold) and underlying clinical evidence of other organ involvement (dry mouth) would have justified a steroid trial. Given that AIP responds dramatically to steroid treatment [13, 17], it is likely that the operation has been avoided in this patient. In patient no. 3, according to the ICDC, the typical imaging would have justified an ampullary biopsy. It is possible that a finding of typical pathology would have prevented preoperative misdiagnosis and unnecessary resection in this case. However, neither of these patients received a steroid trial or a mass biopsy. The other three patients' resections were inevitable, even retrospectively.

The preoperative workup was unsatisfactory for patients scheduled for PDs. The most important clinical parameter is IgG_4_ detection, indicating a biopsy for AIP [18, 19]. But among these five patients, IgG4 was detected only after the operation, with only one patient having a high level. It is possible that IgG4 may have been elevated preoperatively but decreased after surgery. Whether to routinely test patients scheduled for PDs for IgG_4_ remains a big problem. Based on our experience, if CA19-9 is not too high, IgG_4_ detection could be indicated. Only one patient with intermediate imaging was prepared for biopsy, with negative results. Notwithstanding, a biopsy is still recommended [20, 21].

After Sarles et al. [22] described an autoimmune phenomenon in relation to sclerosis of the pancreas in 1961, the concept of AIP was first proposed in 1996 by Yoshida et al. [23]. Since then, AIP has been increasingly documented and is now recognized as an autoimmune disease and a distinct form of pancreatitis, with two types: lymphoplasmacytic sclerosing pancreatitis (LPSP, type 1 AIP) and idiopathic duct-centric pancreatitis (IDCP, type 2 AIP) [24]. Despite the great progress made in the past two decades, the pathophysiology, diagnosis, and treatment of AIP remain a large challenge.

Clinically, AIP usually manifests as painless obstructive jaundice with or without a pancreatic mass, making it difficult to distinguish from pancreatic carcinoma in some cases. As AIP has a dramatic response to steroid therapy, an inaccurate preoperative diagnosis may lead to unnecessary resection. Even though various diagnostic criteria for AIP have been proposed, the incidence (2.5–3.8%) of AIP in patients who undergo PD has not declined over time [7–9]. In the current study, the prevalence of AIP was 4.0% in all PDs performed for presumed malignancy.

The Asian, HISORt, and ICDC criteria are the most important among the various diagnostic criteria of AIP proposed. The Asian diagnostic criteria [15] are consensus criteria established by Japanese and Korean groups. Imaging based on endoscopic retrograde cholangiopancreatography features is mandatory for the diagnosis of AIP. Diagnostic pathology, serology, and response to steroid therapy are included as collateral evidence. Evidence of other organ involvement is not included in these criteria.

The revised HISORt criteria [25] include features highly suggestive of both AIP and PaC and the use of CT as the first step in stratifying patients into three groups, respectively, disease highly suggestive of AIP, indeterminate disease, and disease highly suggestive of PaC. On the basis of CT features, the use of collateral evidence of elevated serum IgG_4_ and other organ involvement can diagnose 70% of AIP patients. However, 30% of AIP patients will require a steroid trial or pathology workup for diagnosis.

The international consensus diagnostic criteria (ICDC) [10] are consensus criteria developed by a panel of Eastern and Western experts after reviewing existing criteria from Japan (2002, 2006) [26, 27], Italy (2003) [28], the United States (HISORt criteria) (2006, 2009) [25, 29], Korea (2007) [30], Asia (2008) [15], and Mannheim (2009) [31]. They encompass two distinct sets of diagnostic criteria for two types of AIP. For AIP of type 1, diagnostic imaging, pathology, serology, other organ involvement, and response to steroids are included. For type 2, diagnostic imaging and pathology, other organ involvement, and response to steroids, but not serology, are included.

AIP patients were misdiagnosed for several reasons. The first is the unsatisfactory preoperative workup, reflecting surgeons' insufficient awareness of AIP. None of the five patients were tested for IgG_4_ or autoantibodies (rheumatoid factor, antinuclear antibodies) before surgery. Nevertheless, serology is one of the most important elements in AIP diagnosis in every existing set of diagnostic criteria. Second, imaging characteristics and other organ involvement in AIP have been overlooked. A diffusely enlarged gland (a landmark feature of AIP) and other organ involvement (a key diagnostic parameter in both the HISORt and ICDC criteria) have not received due attention and have not been further investigated in AIP. Third are the limitations of the current diagnostic strategies. For instance, the HISORt criteria are reportedly capable of diagnosing 70% of AIP patients, but the remaining 30% require a steroid trial or pathology workup for diagnosis [25]. In the current study, even though preoperative workup was sufficient, 3/5 (60%) cases still could not be diagnosed with AIP before surgery.

Obtaining a preoperative pathological diagnosis was a very important issue. Recently, several studies reported the possibility of obtaining a preoperative diagnosis using endoscopic ultrasound-guided fine-needle biopsy [32], using end-cutting needles [33–35], as well as suction technique [36, 37], and applying specific pathologic criteria [38]. However, in our study, we mainly focused on the patients who were initially diagnosed as having malignancies. For pancreatic cancer, if R0 resection can be attainable, pathological diagnosis is not mandatory as long as the diagnostic basis (clinical, laboratory examination, and imaging) is sufficient. If there are uncertainties regarding the diagnosis, especially in distinguishing it from AIP, preoperative pathology becomes crucial. Moreover, if AIP is suspected, adhering closely to diagnostic guidelines for further examination is beneficial.

This analysis is limited due to the retrospective design and the small number of cases. Our hospital is a new center, and most of the referred patients were relatively difficult cases, possibly biasing our candidate selection. In the future, a large number of PD patients should be studied, with much longer follow-ups. As for AIP, more precise diagnostic strategies should be defined and strictly followed.

## 5. Conclusions

AIP usually manifests as painless obstructive jaundice with or without a pancreatic mass, making it difficult to distinguish from pancreatic carcinoma in some cases. The prevalence of benign disease in patients who underwent PD for presumed malignancy was 4.8%, and AIP accounted for 83.3% of these patients. Misdiagnosis of AIP is most likely due to insufficient preoperative workup. IgG_4_ measurement and integrated use of major diagnostic criteria should be emphasized in every patient eligible for PD. Among the patients in this study, 40% of resections could have been prevented in AIP patients scheduled for PD, whereas 60% of AIP patients still cannot be correctly diagnosed before surgery using any current diagnostic strategies. Multiple strategies are needed for preoperative workup.

## Figures and Tables

**Table 1:** Demographic and clinical characteristics of patients with AIP.

Patient no.	1	2	3	4	5
Age	86	53	60	36	68
Gender	M	M	M	F	M
Jaundice	+	+	+	+	+
Pain	-	-	-	+	-
Weight loss	-	+	+	-	-
Diabetes	-	-	-	-	+
Chronic pancreatitis	-	-	-	-	-
Autoimmune disease	+	-	-	-	-
Smoking	-	+	-	-	-
Drinking	-	-	-	-	-

**Table 2:** Imaging characteristics of patients with AIP.

Patient no.	1	2	3	4	5
Diffusely enlarged gland	-	-	+	+	-
Capsule-like rim	-	-	-	-	-
Focally enlarged gland	+	+	-	-	-
Low-density mass	-	-	-	+	+
Pancreatic ductal dilatation (≥3 mm)	-	+	-	-	-
Pancreatic duct cutoff	-	-	-	-	-
Upstream parenchymal atrophy	-	+	-	-	-
Bile duct incrassation (≥3 mm)	+	+	-	-	+
Bile duct stricture	+	+	-	-	-
Radiologic diagnosis	dCCA	dCCA	Ampullary carcinoma	NET	PDAC

dCCA: distal cholangiocarcinoma; NET: neuroendocrine tumor; PaC: pancreatic ductal adenocarcinoma.

**Table 3:** Clinical characteristics of patients with AIP and diagnosis criteria for AIP.

Patient no.	Pathology (preop/postop)	Typical imaging (indeterminate)	CA19-9	IgG	IgG4	AAB	OOI (symptoms)	Steroid trial	Asian criteria	HISORt criteria	ICDC	Biopsy
1	n.a./AIP type 1	No (focally enlarged gland)	30.57	30.61	>3.71	+	No (dry mouth)	No	No	Possible	Possible	No
2	n.a./AIP type 1	No (focally enlarged gland)	147.11	n.a.	n.a.	n.a.	No	No	No	No	No	No
3	n.a./AIP type 1	Yes (seemingly diffusely enlarged gland	n.a.	n.a.	1.26	n.a.	No	No	No	No	Possible	No
4	NET/AIP type 1	Indeterminate (diffusely enlarged gland with low-density mass)	9.33	n.a.	0.55	-	No	No	No	No	No	Negative^∗^
5	n.a./AIP type 1	No	7.45	n.a.	0.51	n.a.	No	No	No	No	No	No

CA19-9 normal < 37 U/ml, IgG < 17 g/L, IgG4 normal < 2.01 g/L. n.a.: not available; AAB: autoantibodies (RF, ANA). ^∗^The biopsy showed something like a neuroendocrine tumor.

## Data Availability

The datasets used and/or analyzed during the current study are available from the corresponding author on reasonable request.

## Abbreviations

AIP: Autoimmune pancreatitis
PD: Pancreaticoduodenectomy
PaC: Pancreatic cancer
Ig: Immunoglobulin
HPF: High power field
SMA: Superior mesenteric artery
PV: Portal vein
SMV: Superior mesenteric vein.
